# Effect of Ester Moiety on Structural Properties of Binary Mixed Monolayers of Alpha-Tocopherol Derivatives with DPPC

**DOI:** 10.3390/molecules27154670

**Published:** 2022-07-22

**Authors:** Grażyna Neunert, Robert Hertmanowski, Stanislaw Witkowski, Krzysztof Polewski

**Affiliations:** 1 Department of Physics and Biophysics, Faculty of Food Science and Nutrition, Poznan University of Life Sciences, Wojska Polskiego 38/42, 60-637 Poznan, Poland; krzysztof.polewski@up.poznan.pl; 2 Institute of Materials Research and Quantum Engineering, Faculty of Materials Engineering and Technical Physics, Poznan University of Technology, Piotrowo 3, 60-965 Poznan, Poland; robert.hermanowski@put.poznan.pl; 3 Faculty of Chemistry, University of Bialystok, Ciolkowskiego 1K, 15-245 Bialystok, Poland; wit@uwb.edu.pl

**Keywords:** tocopherol derivatives, carbo tocopherol, DPPC mixed monolayers, Gibbs energy of excess area and mixing

## Abstract

Phospholipid membranes are ubiquitous components of cells involved in physiological processes; thus, knowledge regarding their interactions with other molecules, including tocopherol ester derivatives, is of great importance. The surface pressure–area isotherms of pure α-tocopherol (Toc) and its derivatives (oxalate (OT), malonate (MT), succinate (ST), and carbo analog (CT)) were studied in Langmuir monolayers in order to evaluate phase formation, compressibility, packing, and ordering. The isotherms and compressibility results indicate that, under pressure, the ester derivatives and CT are able to form two-dimensional liquid-condensed (LC) ordered structures with collapse pressures ranging from 27 mN/m for CT to 44 mN/m for OT. Next, the effect of length of ester moiety on the surface behavior of DPPC/Toc derivatives’ binary monolayers at air–water interface was investigated. The average molecular area, elastic modulus, compressibility, and miscibility were calculated as a function of molar fraction of derivatives. Increasing the presence of Toc derivatives in DPPC monolayer induces expansion of isotherms, increased monolayer elasticity, interrupted packing, and lowered ordering in monolayer, leading to its fluidization. Decreasing collapse pressure with increasing molar ratio of derivatives indicates on the miscibility of Toc esters in DPPC monolayer. The interactions between components were analyzed using additivity rule and thermodynamic calculations of excess and total Gibbs energy of mixing. Calculated excess area and Gibbs energy indicated repulsion between components, confirming their partial mixing. In summary, the mechanism of the observed phenomena is mainly connected with interactions of ionized carboxyl groups of ester moieties with DPPC headgroup moieties where formed conformations perturb alignment of acyl chains, resulting in increasing mean area per molecule, leading to disordering and fluidization of mixed monolayer.

## 1. Introduction

Phospholipids are vital components of cells fulfilling their structural and functional roles. Among them, phosphatidylcholine DPPC is one of the most ubiquitous lipids in living organisms; thus, its investigations are carried out in many fields of biology, pharmacology, and medicine, using them as liposomes or in the form of monolayer. Properties of this last example are of particular interest because they are used as a model of natural surfactant systems present in lung surfactant, eyes, or ears, especially regulation of surface tension at air–liquid interface in lung alveoli. Phospholipid monolayers are also of great interest in pharmacology as possible efficient carriers for drug delivery.

The role of α-tocopherol (Toc) in cells is mainly connected with its antioxidant protection; however, much evidence has also shown that the presence of Toc in biological membranes, even in low concentration, influences on phase behavior of phospholipid bilayers in membranes [1,2,3,4] and simultaneously stabilizes the structure of membranes [5]. Such stabilization arises from interactions between phytyl chain of Toc and acyl chains of phospholipids, with some minor participation of interactions between chromanol ring and hydrophilic groups of phospholipids. The presence of hydroxyl group makes this compound an amphiphilic molecule able to form stable monolayers and simultaneously able to penetrate into the interior of the membrane. However, the miscibility of Toc in the membrane is limited to a few mole fraction and, in higher Toc concentrations, destabilization of the membrane structure occurs [6]. The surface properties of Toc have been described in a few papers [6,7,8]. Transition from 3D structures as liposomes to 2D monolayers as a model of half of a bilayer allows better control of the monolayer through its organization and interactions between phospholipids and other components [9].

In the search to improve some limiting tocopherol properties, new tocopherol derivatives were synthesized and investigated. It appeared that chemical composition of derivatives influences their properties, especially presence of different moieties at the chromanol ring. Some of them, such as tocopherol acetate, tocopherol succinate (ST), or its water-soluble form, an alpha-tocopheryl polyethylene glycol succinate (TPGS), are already used as drugs. There is a group of derivatives substituted at 6-position of the chromanol ring, including esters, such as oxalate, malonate, and succinate, or substituted with glycosides, tocopheryl nicotinate or linoleate. Substitution at 6-position devoid this molecule an antioxidant property; however, its presence in model membranes or in cells influences the ordering and structural properties of membranes [10,11,12,13].

Lipid monolayers serve as a simple model of biological membranes and are used to evaluate membrane behavior at a molecular level with inserted ligands as, for example, drugs, cholesterol, or tocopherols [6,14,15,16]. Relatively simple measurements of compression isotherms give insight into conformational order and intermolecular interactions of molecules at the monolayer interface. Using this methodology in this study, we will test the hypothesis that the presence of attached ester moieties at 6-position in the chromanol ring of tocopherol influences the stability and structure of biological membranes and attempt to characterize the molecular interactions’ mechanism governing the observed phenomena. According to our knowledge, such systematic studies of the binary DPPC/ester derivative monolayers have not been reported.

**Figure 1:**
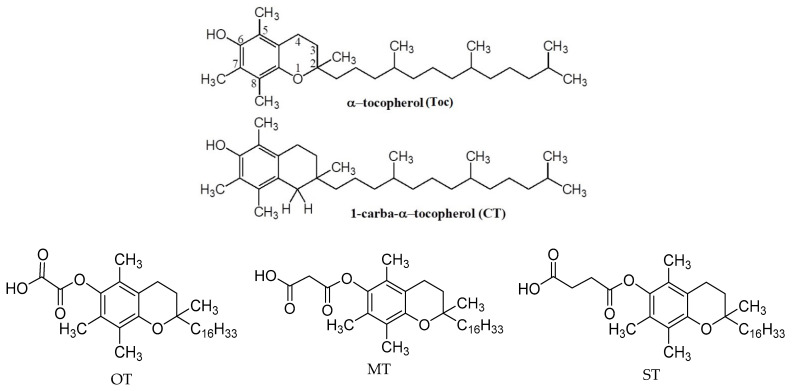
Chemical structures of α-tocopherol (Toc) and its derivatives: 1-carba-α-tocopherol (CT), DL-α-Tocopheryl oxalate (OT), DL-α-Tocopheryl malonate (MT), and DL-α-Tocopheryl succinate (ST).

In these studies, the role of ester groups attached at 6-position of the chromanol ring of Toc on structural properties of mixed monolayers of Toc derivatives with DPPC has been investigated. The studied derivatives included esters (oxalate OT, malonate MT, and succinate ST) and, additionally, a carbo analog of Toc (CT) devoid of an oxygen atom at 1-position in the chromanol ring and replaced by a methylene group (Figure 1).

## 2. Results

During experiments, the surface pressure and compressibility isotherms of tocopherols and DPPC mixed monolayers versus mean molecular area per molecule with increasing presence of pure Toc and its derivatives were recorded. The obtained values were plotted as function of molar composition of monolayer and compared with those obtained from theoretical calculations according to the additive rule for ideally mixed films [17]. To determine the possible type of interactions and monolayer stability, the thermodynamic properties of mixed monolayers were calculated, including the Gibbs energy of excess and total energy of mixing. The first part of the studies was devoted to characterization of monolayer properties of pure Toc and its derivatives. In the second part, the properties of binary mixture of DPPC/derivatives were investigated.

To estimate the influence of the electrostatic charges on Toc behavior, we carried out the isotherm measurements at neutral (pH 7) and acidic (pH 3) subphase. Obtained results delivered additional information regarding biophysical properties of Toc molecule in the membranes.

### 2.1. Monolayers of Pure Tocopherol and Its Derivatives

#### 2.1.1. π-A Isotherms

**Figure 2:**
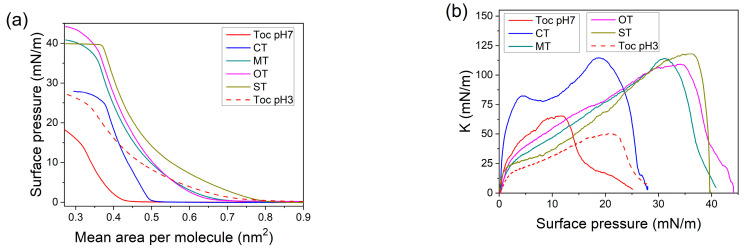
Surface pressure—area (π-A) (**a**) and compressibility modulus–surface pressure (K-π) (**b**) isotherms for Toc, CT, and esters (OT, MT, and ST).

The surface pressure–mean area per molecule (π-A) isotherms of Toc and its derivatives are presented in Figure 2a. It shows that pure Toc and its derivatives, when spread onto water subphase, form compressible monolayers which exhibit different isotherm shapes. The lowest collapse pressure (π_c_) is observed for Toc and CT, molecules having OH moiety at 6-position of the chromanol ring. The three ester derivatives have higher π_c_ than Toc however, its value decreases with increasing length of attached moiety. It is also visible from Figure 2a that, above surface pressure of 5 mN/m, the isotherms of esters are expanded compared to Toc. Increasing onset area (A_1_)—a value at which the surface pressure is detected—indicates the presence of charged moieties attached to the chromanol ring of derivatives causes repulsion that decreases packing density and cohesive interactions between molecules. Such behavior is observed with ionic ester derivatives and Toc at pH 3. Isotherm expansion may also reflect the presence of steric hindrance between molecules, as was shown for dl-α-tocopheryl β-Dglucopyranoside derivative [12].

A CT isotherm is located between esters and Toc with π_c_ at 27 mN/m which occurs at 0.39 nm^2^ with further plateau. The small value of A_1_, π_c_, and shape of CT isotherm indicates that the molecule, during its interaction with subphase, has a tendency to stay in a less ordered gas phase than Toc.

We may notice that shapes of isotherms for Toc at pH 7 and pH 3 differ. The former has onset at a much lower area than that at pH 3, lower π_c_, and the lowest limiting surface area (A_t_), obtained from extrapolation to zero surface pressure of the straight part of isotherm, assuming the most packed phase. This shape of Toc at neutral subphase is similar to that reported by others [6].

#### 2.1.2. Compressibility Analysis

Under compression, the monolayer adopts various physical states, which depend on the level of conformational order of the molecules at the interface and the presence of intermolecular interactions within the monolayer [18]. At the beginning of compression at high molecular areas, there can be a gaseous phase in which molecules are widely separated. In next step, liquid expanded (LE) state, where molecules are closer together and ordered like a two-dimensional liquid, is observed. Further compression causes the appearance of liquid condensed (LC) state, in which the aliphatic chains are densely packed. At the end, at very high surface pressures, two-dimensional solid-like state can be observed for some compounds. Co-existence regions between phases are often encountered, such as LE/LC mixed phase, in which dispersion of LC islands as a superlattice in a continuous phase of LE molecules is observed [19]. The assignment of the physical state of monolayer is based on Davies and Rideal classification [20]. Surface compressibility modulus (K) gives opportunity to detailed characterization of phase changes and elasticity, which are related to packing density and/or ordering of monolayers and was calculated according to Equation (1) [20]:

(1) $$K=-A\frac{\partial \pi }{\partial A}$$

Figure 2b presents compressibility–surface pressure (K-π) plots of Toc and its investigated derivatives. In each case, we observe continuous transition from gaseous state into liquid-expanded (LE) state at the surface pressure in the range of 0.5 mN/m. Further pressure increase is leading to continuous formation of LE phase and, in the case of CT, OT, MT, and ST, a transition to mixed LE and liquid-condensed (LC) phase is also observed. Ester derivatives up to 30 mN/m exist as a flexible monolayer in LE/LC mixed phase, whereas, above that surface pressure, they exhibit compressibility values above 100 mN/m, which indicates their transition into LC phase following sharp collapse at about 40 mN/m. The π-A isotherms for Toc and CT show quite similar low values of collapse pressure, Figure 2a, whereas the compressibility plots show significant differences. For Toc, a compression isotherm shows that monolayer exists in LE fluid state in a full range of surface pressures. CT at low pressure exists in LE state and easily forms mixed LE/LC phase. The minimum of compressibility isotherm at about 8 mN/m indicates its phase transition to LC state. With increasing surface pressure, immediately before collapse, it reaches a maximum value of compressibility modulus of 120 mN/m at 20 mN/m of surface pressure. It shows that, for CT with this same hydroxyl group at 6-position as Toc but with the presence of a methyl group at 1-position of the chromanol ring, the changes introduced by such substitution rearranged the electronic distribution in the chromanol ring, allowing for the formation of LC state not reachable for Toc. Presented above, isotherms and compressibility results indicate that, under pressure, the investigated molecules are able to form a two-dimensional ordered structure.

### 2.2. Mixed Monolayers of DPPC with Tocopherol and Its Derivatives

#### 2.2.1. π-A Isotherms of Mixed Monolayers

**Figure 3:**
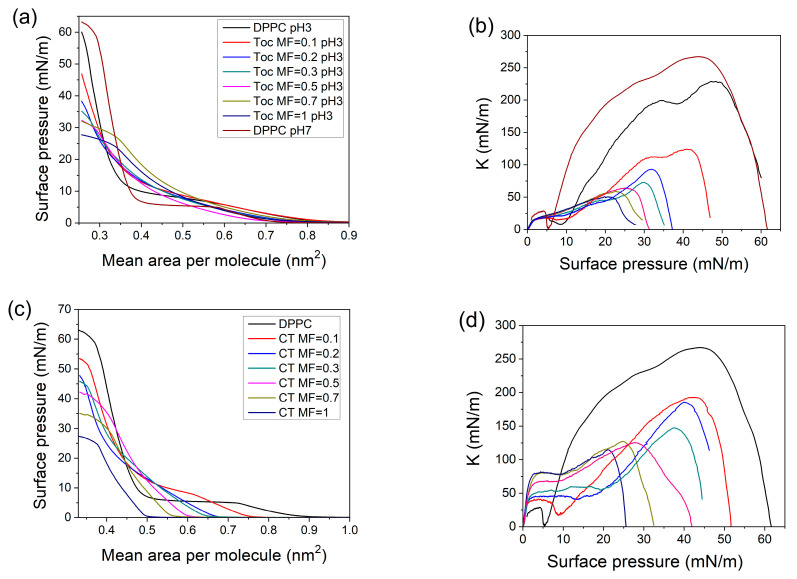

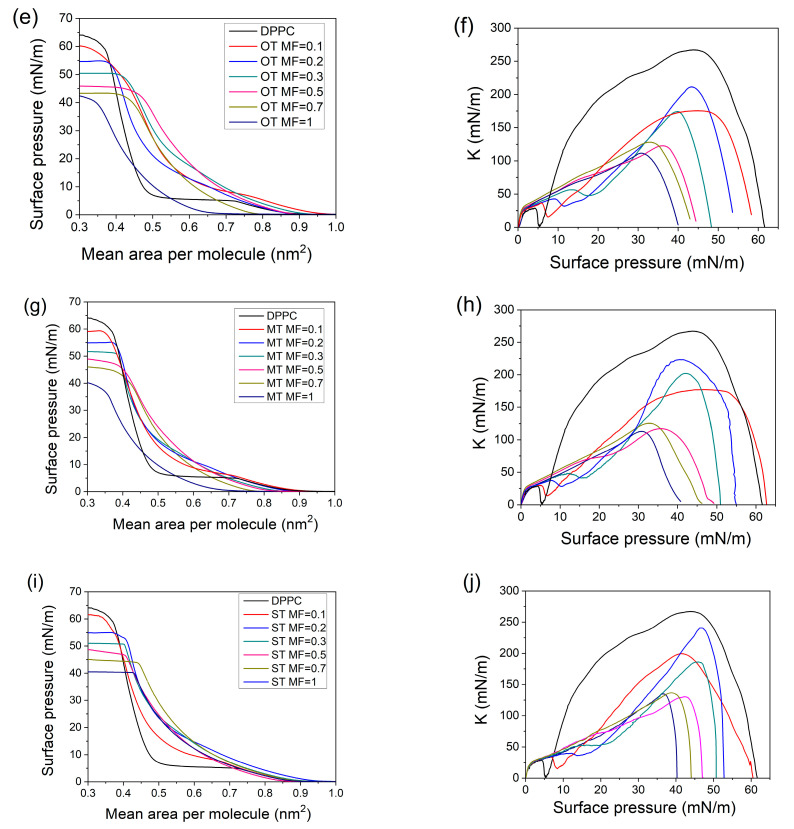
Surface pressure–mean molecular area (π-A) (**a**,**c**,**e**,**g**,**i**) and compressibility (K-π) (**b**,**d**,**f**,**h**,**j**) isotherms for mixture of DPPC and: (**a**,**b**) Toc; (**c**,**d**) CT; (**e**,**f**) OT; (**g**,**h**) MT; (**i**,**j**) ST. Molecular fraction (MF) of components is given in the legend. All π-A and K-π isotherms for different MF of Toc were obtained at pH 3. All presented plots for CT and investigated esters were collected at pH 7.

The effects of the presence of Toc and its derivatives in DPPC monolayer on recorded π-A and compressibility isotherms are illustrated in Figure 3. The shape of pure DPPC isotherm on subphase at pH 3 is very close to that recorded at pH 7, with its characteristic broad plateau at almost constant surface pressure of 8 mN/m ascribed to LE/LC equilibrium and π_c_ at about 60 mN/m being consistent with results obtained in this work and reported by others [15].

The π-A isotherms of mixed DPPC/Toc monolayers at pH 3 at different Toc molar fractions are shown in Figure 3a. The A_1_ parameter slowly changes with Toc concentration. At surface pressure below 7 mN/m, the increasing presence of Toc leads to contraction of isotherms, whereas, above that pressure, an expansion of isotherms occurs. Simultaneously, increasing molar ratio of Toc leads to lowering of π_c_ of such mixtures. The expansion shapes of isotherms for 50 and 70 mol% of Toc differ compared to those at lower concentrations; however, they show close π_c_ values. The isotherm for 70 mol% displays a very similar shape as that for pure Toc, thus reflecting the dominant presence of Toc in the sample. Elevated π_c_ compared to pure Toc suggests interaction between components.

The increasing presence of ester derivatives in DPPC at low surface pressure shows that A_1_ for a gas-to-LE phase transition gradually decreases and leads to a disappearance of plateau observed for DPPC. Up to 8 mN/m, the π-A isotherms of mixed monolayers exhibit compression, whereas, above that surface pressure, the expansion of isotherms is observed. The shapes of isotherms change, showing increasing surface pressure corresponding to onset of LC transition with further reaching π_c_. At concentrations above 30 mol%, the isotherms approach the shapes observed for pure ester derivatives (Figure 3e,g,i). With increasing concentration of each derivative, the π_c_ decreases from the highest value of 65 mN/m for DPPC to lowest value of 40 mN/m observed in ester derivatives.

The increasing presence of CT in DPPC shows, as seen in Figure 3c, that onset point to LE phase transition correspondingly shifts to lower values of area per molecule, indicating that, in mixed monolayers, a gas phase with LE coexistence region (at zero surface pressure) is significantly lowered. Such regularity is observed till a surface pressure of 11 mN/m. With increasing compression and CT concentration, the expansion of isotherms is observed, with simultaneous disappearing of the equilibrium plateau of LE/LC mixed state and increasing surface pressure corresponding to onset to LC transition being observed. At concentrations of 50 and 70 mol%, the isotherms approach shapes of pure CT, with simultaneous decreasing of π_c_ and showing formation of LC phase before collapse. Proportional lowering of π_c_ observed with increasing content of CT indicates its efficient mixing with DPPC molecules.

#### 2.2.2. Compressibility Analysis

The state of monolayer, including phase transition, packing order, and more precise determination of its π_c_, are better determined with K-π plots, as given in Figure 3b,d,f,h,j.

For pure DPPC monolayer at pH 3 at surface pressure of 20 mN/m, K values are above 100 mN/m, with a maximum of around 250 mN/m, which indicates its transition into LC state where forming of condensed highly packed films occurs. At lower surface pressure, it shows a characteristic dip at about 8 mN/m, indicating LE/LC phase transition.

The presence of increasing amounts of Toc leads to changes in shape of compressibility curves in mixed monolayers. In the whole range of Toc concentrations, except at 10 mol%, the absence of LE/LC phase transition is observed. The obtained K maxima are below 100 mN/m, indicating that increasing presence of Toc shifted monolayers to LE phase, forming the more compressible and less dense monolayers.

The increasing amount of CT in mixture correspondingly lowers the maximum of K; however, above 20 mN/m of surface pressure at all concentrations, the mixed monolayers remain in LC phase. The surface pressure corresponding to the minimum compressibility isotherm increases, with a CT concentration up to 30 mol% and indicating the formation of equilibrated LE/LC mixed phase, as observed in corresponding π-A isotherms (Figure 3c). The minimum located at 8 mN/m observed for pure CT and 50 and 70 mol% suggests that the monolayer is in liquid state.

All investigated esters show a similar pattern of behavior where, up to 30 mol%, the LE/LC phase transition at increasing surface pressure and compressibility occurs. The K maximum for 10 mol% at surface pressure of 38 mN/m is 150 mN/m, whereas, for 20 ad 30 mol% at surface pressure of 45 mN/m, the maximum increases to 200 and 175 mN/m, respectively. The presence of 50 and 70 mol% esters in mixed monolayers lowers maxima to the range of 120 mN/m, indicating that it still exists in LC phase, however, with progressively decreasing surface pressure at collapse and with a shape very similar to pure esters. Before collapse in the whole range of concentrations, the mixed monolayers exist in LC state with consecutive lowering of surface pressure at collapse.

#### 2.2.3. Miscibility Analysis and Thermodynamics of Mixed Monolayers

The miscibility analysis and determination of types of possible interactions between components in mixed monolayer was based on additivity rule and was estimated from an analysis of excess area per molecule (ΔA_exc_), defined by Equation (2), as the function of molar fractions of components at selected surface pressures [21]:

ΔA_exc_ = A_12_ − (A_1_·MF_1_ + A_2_·MF_2_) (2)

where A_12_, A_1_, and A_2_ are the mean areas per molecule for mixed monolayer and single-component monolayers, respectively, determined at the same surface pressure, and MF_1_ and MF_2_ are the mole fractions of the components.

**Figure 4:**
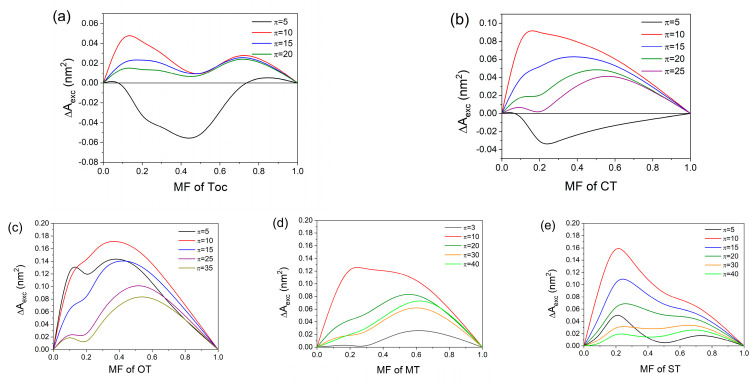
Excess area (ΔA_exc_) versus mole fraction (MF) of (**a**) Toc, (**b**) CT, (**c**) OT, (**d**) MT, and (**e**) ST, obtained at different surface pressure (mN/m), as shown in legend.

When ΔA_exc_ = 0, both components are immiscible or totally miscible. In cases when ΔA_exc_ is different from 0, it indicates partial miscibility of the components but with different interaction mechanisms, where negative values indicate attractive forces and positive values indicate repulsive forces between components. The ΔA_exc_ values as a function of mole fraction of investigated derivatives at different surface pressures are shown in Figure 4. To assure the presence of both components in monolayer, the chosen surface pressures are lower than collapse pressure of the investigated derivative.

It shows that, for Toc and CT, at pressures below 10 mN/m, a negative sign of ΔA_exc_ is observed, indicating the presence of attractive interactions. From the value of 10 mN/m in the whole range of concentrations, the positive values of ΔA_exc_ are observed, indicating repulsive interactions between components.

The investigated esters in the whole range of concentrations and surface pressures exhibit a positive sign of ΔA_exc_, which indicates that the area occupied by mixed monolayer is larger than the area occupied by the same amount of separate components, which indicates the appearance of a repulsive interaction between molecules and only partial miscibility of components in the monolayer. For all tocopherols, the highest ΔA_exc_ values are observed at a surface pressure of 10 mN/m at the mole fraction of 0.2–0.3, whereas further pressure increase diminishes ΔA_exc_.

To quantify the molecular interactions between components and determine magnitude of those interactions, we apply thermodynamic analysis by calculating the excess Gibbs energy of mixing ΔG_exc_ according to Equation (3) [21,22]:

(3) $$∆G_{\mathrm{exc}}=N\overset{\pi }{\underset{0}{\int }}A_{\mathrm{exc}}d\pi$$

where N is the Avogadro number.

The thermodynamic stability of the system was determined based on the total free Gibbs energy of mixing ΔG_mix_ from Equation (4) [6,21]:

(4) $$∆G_{\mathrm{mix}}=∆G_{\mathrm{exc}}+\mathrm{RT}\left({\mathrm{MF}}_{1}{\mathrm{lnMF}}_{1}+{\mathrm{MF}}_{2}{\mathrm{lnMF}}_{2}\right)$$

where R is the gas constant and T is the temperature. A negative sign of ΔG_mix_ indicates monolayer stability, whereas positive values suggest phase separation. For ideal mixing or total immiscibility, the ΔG_mix_ equals zero.

**Figure 5:**
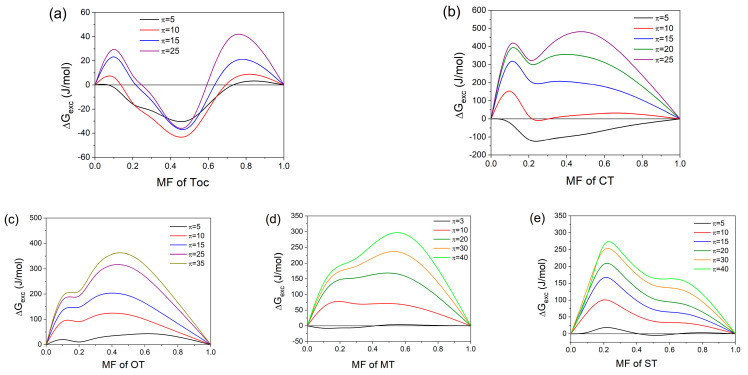
The excess free energy of mixing ΔG_exc_ versus mole fraction of (**a**) Toc, (**b**) CT, (**c**) OT, (**d**) MT, and (**e**) ST as a function of monolayer composition at different surface pressure (mN/m), as shown in legend.

**Figure 6:**
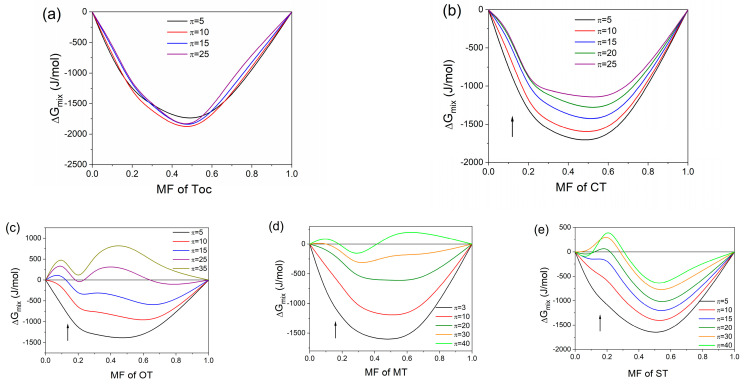
The total free energy of mixing ΔG_mix_ versus mole fraction of (**a**) Toc, (**b**) CT, (**c**) OT, (**d**) MT, and (**e**) ST as a function of monolayer composition at different surface pressure, as shown in legend.

The results obtained for ΔG_exc_ are presented in Figure 5. It shows that the nature of interaction and its magnitude depends on the molar fraction of components, surface pressure of monolayers, and type of component. For Toc, the most attractive interactions occur only at MF = 0.5 in the whole range of surface pressures, whereas, at MF = 0.1 and 0.8, two maxima with positive energies increasing with rising surface pressure are observed. For CT, attractive interactions are present in the whole range of concentrations only at a surface pressure of 5 mN/m. The increasing surface pressures lead to increasing of positive energy, with the formation of two maxima at molar fractions of 0.1 and 0.5. For esters, at low surface pressures, the excess energies are in the range of zero, indicating mixing of components. Above 10 mN/m, the repulsive interactions are increasing, showing maxima at MF = 0.1 and 0.5. Negative values of ΔA_exc_ and ΔG_exc_ obtained for Toc at MF = 0.5 indicate the formation of the most preferred monolayer structure at 1:1 molar ratios, which is confirmed by negative ΔG_mix_ values, as seen in Figure 6a, which is indicative of its thermodynamic stability. Observed positive values indicate repulsive forces between components that can lead to phase separation or partial mixing of the components in mixed monolayers. The presence of maxima separated by a minimum suggests formation regions enriched in one of the components, giving rise to partial miscibility.

The ΔG_mix_ results for Toc derivatives are shown in Figure 6b–e as a function of monolayer composition at surface pressures. Obtained negative values ΔG_mix_ for CT indicate a monolayer stability; however, contrary to Toc where those values remain nearly constant at investigated surface pressures, for CT, it decreases fast with increasing surface pressure. Ester derivatives exhibit the highest stability at lower surface pressures, being most stable for ST derivative. Increasing surface pressure lowers stability in the whole range of concentrations. For ST, MT, and OT, the ΔG_mix_ values are close to zero or show positive values, making the mixture less stable. Especially for OT at surface pressures above 20 mN/m, the monolayer becomes unstable, suggesting that increasing compressibility introduces repulsion between molecules due to steric hindrance.

## 3. Discussion

In our previous studies, we have found that the presence of ester derivatives, OT and ST, or CT causes a disruptive effect in DPPC liposomes [10,11,13]. To add more knowledge regarding the molecular mechanisms of the interactions between phospholipids and tocopherol derivatives, the studies in mixed Langmuir DPPC/Toc esters monolayers were carried out. Obtained information on miscibility, packing order, interactions, and thermodynamic parameters of interacting components allowed the addition of new information on tocopherol esters’ behavior in biological membranes. Quantum chemical calculation of electrostatic potential surface of Toc molecule has shown that most regions of that molecule is nearly neutral, except the regions of two oxygen atoms and a phenolic hydrogen atom that suggest that possible interactions at those places may lead to modified behavior of tocopherols [23]. To widen the range of discussion regarding the possible mechanism of interactions, we included in our consideration the results obtained for Toc at pH 3 and for CT.

**Figure 7:**
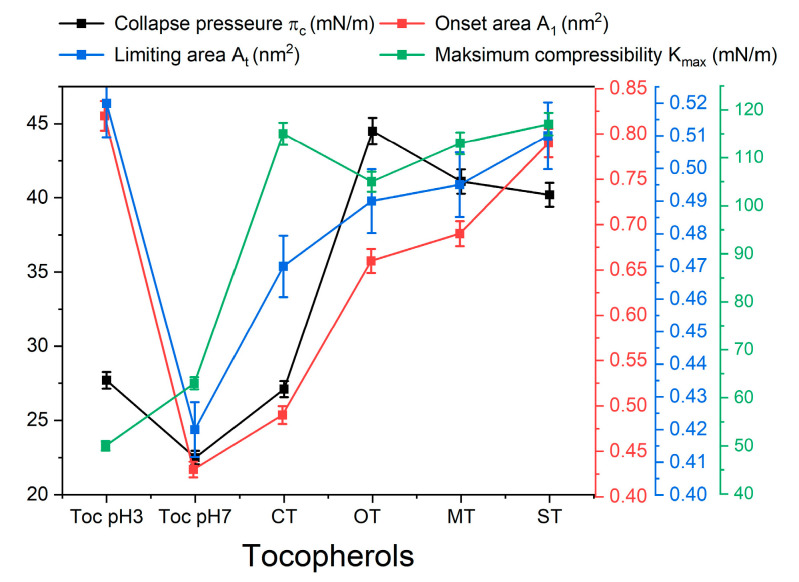
Parameters, given in the legend, calculated from isotherms presented in Figure 2. Error bars are SD calculated for three different repetitions.

To estimate possible relations between tocopherols, the parameters, such as A_1_, A_t_ π_c_, and maximum compressibility value K_max_, calculated based on measured isotherms, were plotted in Figure 7.

The data obtained for both tocopherols confirmed that they form less stable monolayers in LE phase, with low compressibility modulus. Very probably, it is connected with the presence of R and S stereoisomers, which create steric hindrance to form more dense monolayers. The differences in shapes of isotherms and compressibility plots measured at neutral pH and at pH 3, as seen in Figure 2, are clearly visible, as well as the calculated parameters (Figure 7). The observed differences between isotherms from a chemical point of view may be explained by oxidation or by ionization. According to results obtained from constant π–A studies, the expansion of isotherms is caused by ionization [8]. Toc at pH 3 also exists only in LE state and its lift-off area is much larger than that for neutral Toc. This indicates that the presence of protonated Toc, mostly at the chromanol ring, induces electrostatic repulsions between molecules, leading to its poor packing density in formed films. Taking into account the above presented data, we suggest that, in such simple systems, the ionization process of Toc might be responsible for the obtained results. Of course, this does not exclude any other possible mechanism; however, the smooth shape of isotherm does not provide an assumption of any other processes.

In the case of CT, the replacement of O1 in the chromanol ring by methylene group leads to its different properties compared to parent compound. The observed dip at 8 mN/m on the compressibility plot indicates a transition from LE to LC state, thus leading to the formation of much more ordered structure than Toc. In this case, substituted at 1-position of the chromanol ring, a methylene group introduces hydrophobicity to this region and changes electronic distribution, decreasing repulsive interactions between molecules, thus allowing for more condensed and ordered structure.

An important monolayer parameter, such as π_c_, showing low values obtained for Toc and CT suggests that both compounds exhibit a good surface activity, and thus possess the ability to spontaneously penetrate the membrane interface. Such behavior for Toc has been reported for liposomes and biological membranes [24]. In the case of CT, its inclusion into DPPC liposomes has been reported in our studies [13].

The results obtained for the Toc ester derivatives differ between them and also vary from Toc and CT. Since investigated esters differ only in moiety attached at 6-position of the chromanol ring, it is very probable that observed changes are related to physicochemical properties of attached moieties. Such relation is seen on compressibility plots, as shown in Figure 2b, where π_c_ of Toc and its analog CT, having a phenolic group at 6-position, is significantly lower compared to Toc ester derivatives with attached ester moieties. We suggest that such deviation of π_c_ of Toc derivatives compared to Toc is related to interaction of ionizable moieties with aqueous subphase. In fact, all ester derivatives possess one or two hydrophilic carbonyl groups, which may effectively interact with aqueous subphase. In gas phase, the molecules lay flat on the subphase surface, with the ionizing group interacting with aqueous subphase. Increasing compression leads to formation of LE phase, where polar groups interact with aqueous phase, whereas chromanol ring with adjacent fatty acid chain is forced into an upright position and, finally, at pressures before collapse, some ordered structures are formed and, eventually, the transition into a coherent LC monolayer occurs. In the proposed mechanism, we suggest that, for Toc, with increasing surface pressure due to steric hindrance, only phytyl chain may be shifted into an upright position, whereas the chromanol ring still remains flat interacting with aqueous subphase. When the chromanol ring may arrange into an upright position, it is possible that molecules may get closer and start van der Waals interactions between phytyl chains, thus allowing formation of ordered monolayer structures, which occurs in the case of CT and investigated Toc ester derivatives [25].

The A_t_ of DPPC referred to as a cylindrical molecule should be 0.37 nm^2^/molecule, assuming that a single palmitoyl tail occupies 0.18 nm^2^/molecule. However, A_t_ values obtained experimentally are in the range of 0.50 nm^2^/molecule. This arises from the fact that the molecule has to accommodate the head group area, which leads to conformational changes in polar head group and tilting of acyl chains [26,27]. Similar reasoning may be applied to Toc derivatives, which poses one phytyl chain with a limiting area in the range of 0.20 nm^2^/molecule, whereas measured A_t_ are in the range of 0.47–0.50 nm^2^/molecule. Such discrepancy indicates that A_t_ of pure derivatives should also include area of ester moieties lying flat on aqueous subphase.

**Figure 8:**
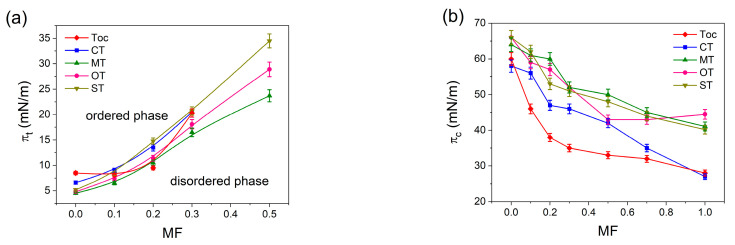
Plots of (**a**) LE/LC transition pressure π_t_ and (**b**) collapse pressures π_c_ for investigated tocopherols, as given in the legend. Error bars are SD calculated for three different repetitions.

The inclusion of Toc ester derivatives with increasing concentrations into DPPC monolayer caused remarkable changes in shapes of isotherms, compressibility plots, and calculated parameters, as presented in Figure 3, Figure 4, Figure 5, Figure 6 and Figure 7. For all ester derivatives, the expansion of isotherms and significant shortening of LE–LC plateau region, which disappears at increasing partition of derivatives, is observed. A characteristic behavior of increasing the presence of ester derivatives in DPPC mixture is lowering of its π_c_, whereas the values of those changes differ depending on the derivative with lowest value recorded for ST (Figure 8b). Moreover, the surface pressure π_t_ corresponding to LE/LC transition increases with added component, which is a typical observation indicating the formation of mixed films by miscible components (Figure 8a). All investigated compounds embedded into DPPC monolayer decreased its stability, as shown by lowered π_c_, and induced an increasingly liquid character. Finally, observed lowering of π_c_ with increasing molar ratio of derivative in monolayer mixture indicates that both components are miscible.

In DPPC monolayer in LE phase, the phospholipid molecules are in the upright or tilted position. With added Toc ester derivatives, the chromanol ring with attached moieties still may lay flat on subphase, whereas increasing surface pressure may lead to vertical orientation of fatty chain and chromanol ring. The presence of an ionizable carbonyl group at the end of moiety may also interact with ionic molecules in the head region of phospholipid, leading to interactions between components. Such types of interaction between phospholipids and embedded molecules have been reported for propranolol embedded into POPC bilayer, where propranolol binds specifically to carbonyl and phosphate groups, resulting in an increase in packing in the polar head group or decrease in lipid tail region [28]. Moreover, the presence of nonselective β-blockers was reported to fluidize DPPC lipid bilayer [29]. During studies of Toc location in saturated membranes and using number of biophysical technics, it is found that the tail chain of Toc lies almost parallel to the acyl chain of DPPC, whereas the hydroxyl group of chromanol ring establishes hydrogen bonding with water and the main axis of Toc molecule is perpendicular to the bilayer plane [30,31].

As shown in Figure 8a, the observed linear increase in π_t_ with rising ester partition indicates that the coexistence region is decreasing. The isotherms obtained for mixed monolayers show that π_t_ varies with composition, which is behavior observed for miscible components interacting on the aqueous subphase. The results obtained from plots of excess area and calculated Gibbs energy of excess confirmed the conclusion that, at low molar ratios, the Toc ester derivatives were miscible in DPPC films. Simultaneously, a slow decrease in π_c_ is observed, which confirms partial miscibility of ester derivatives in DPPC monolayer. The presented results show the tendency of decreasing of the surface compression of DPPC monolayers and making the monolayers more flexible when mixed with investigated Toc derivatives. It means that investigated derivatives introduce loosening of the closely packed structure of pure DPPC.

It is very probable that, in our samples, at higher surface pressures above the stability limit, there will appear 3D structures, including DPPC bilayer; however, during our measurements, we limited surface pressures to first collapse. We consider its linear shape in surface pressure limits as evidence that no bilayer structure has been formed. BAM studies have shown that, close to the end at collapse pressure, Toc is squeezed out from the 0.5/0.5 mixed DPPC/Toc monolayer [6].

Our results indicate that investigated Toc derivatives, when included into biological membranes, at which the surface pressure is about 30–35 mN/m, may modify lateral pressures in biological structures [32]. They may form miscible structures, leading to interactions with phospholipids, which increase membrane fluidity and introduce disorder in membrane structure. Since all of the investigated ester derivatives are not antioxidant, their presence in membrane will always be destructive, as has been shown for ST or OT during its interaction with cell lines [33], cancer cells [34,35], or in DPPC liposomes [10,11]. Moreover, CT, which bears some antioxidant properties when embedded into liposomes, efficiently fluidizes its structure [13].

**Scheme 1:**
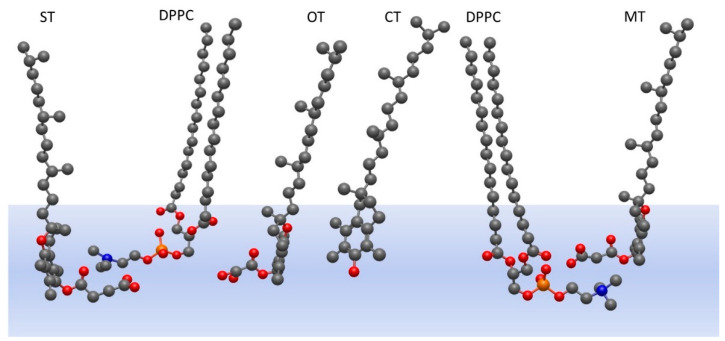
Cartoon of one of the possible alignments of derivatives during interactions with the aqueous subphase.

Scheme 1 visualizes a proposed model of interactions between derivatives and subphase, as was discussed in this work. It presents possible distribution of molecules in the most packaged states of monolayer.

## 4. Materials and Methods

### 4.1. Reagents

1-Carba-α-tocopherol ((2,5,7,8-tetramethyl-2-(4,8,12-timethyltridecyl)-1,2,3,4-tetrahydronaphtalen-6-ol)) (CT), DL-α-Tocopheryl oxalate (OT), DL-α-Tocopheryl malonate (MT), DL-α-Tocopheryl succinate (ST), and DL-α-tocopherol (Toc) were obtained according to previously published procedures [36,37]. 1,2-Dipalmitoyl-sn-glycero-3-phosphocholine (DPPC), chloroform, and methanol (both spectroscopic grade) were purchased from SIGMA Chemical Co. (St. Louis, MO, USA). Double deionized water produced with a MicroPure Water System (Millipore Co., Guyancourt, France) was used as a solvent. The chemical structures of the tocopherol and its derivatives are shown in Figure 1.

### 4.2. Preparation of Langmuir Monolayers

The substances were dissolved in chloroform at a concentration of 1·10^−3^ M to obtain stock solutions. Mixtures of specified mole ratios were prepared from these stock solutions just before being spread. Then, an appropriate amount (30 µL) was deposited drop by drop onto a clean water surface from a microsyringe (Hamilton, Bonaduz, Switzerland). After the solvent was allowed to evaporate (10 min), the films were compressed symmetrically on a Minitrough 2 (KSV Instruments, Helsinki, Finland) at a barrier motion speed of 0.041 nm^2^·min^−1^·molecule^−1^. Ultrapure deionized Milli-Q (Millipore Co., Guyancourt, France) filtered water with a specific resistance of 18.2 MΩ·cm was used as a subphase at a temperature that was kept constant at 20 °C by a cooling circulator F12ED (Julabo, Seelbach, Germany). A platinum Wilhelmy plate hanging from a balance was used to record the surface pressure value.

### 4.3. Data Analysis and Fitting Procedures

All plots, figures, calculations, and fitting procedures, including statistics of the plotted data, were prepared using the Origin program (ver. 8.5, OriginLab Corp., Northampton, MA, USA). All experiments were repeated at least in triplicate. The cartoon at Scheme 1 was prepared using conformation obtained from Avogadro 1.2 version and each molecule corresponds to free molecule.

## 5. Conclusions

Comparative studies on behavior of tocopherol derivatives in Langmuir monolayer have shown that inclusion of ester derivative into DPPC monolayer induces its increasingly liquid expanded character. The mechanism of the observed phenomena is mainly connected with interactions of ionized carboxyl groups of ester moieties with DPPC headgroup molecules, where formed conformations perturb alignment of acyl chains, resulting in increasing mean area per molecule, leading to disordering and fluidization of mixed monolayer. In contrast, Toc easily mixes with DPPC, forming the most stable monolayers and, at low concentrations, only negligibly influences monolayer stability.

## Data Availability

Data sharing not applicable.

## Sample Availability

Samples of the compounds (CT, Toc, OT, MT, and TS) are available from the authors.
